# Nanowrinkled Carbon Aerogels Embedded with FeN_*x*_ Sites as Effective Oxygen Electrodes for Rechargeable Zinc-Air Battery

**DOI:** 10.34133/2019/6813585

**Published:** 2019-12-20

**Authors:** Ting He, Bingzhang Lu, Yang Chen, Yong Wang, Yaqiang Zhang, John L. Davenport, Alan P. Chen, Chih-Wen Pao, Min Liu, Zhifang Sun, Alexander Stram, Alexander Mordaunt, Jairo Velasco, Yuan Ping, Yi Zhang, Shaowei Chen

**Affiliations:** ^1^State Key Laboratory for Powder Metallurgy, College of Chemistry and Chemical Engineering, Central South University, Changsha 410083, China; ^2^Department of Chemistry and Biochemistry, University of California, 1156 High Street, Santa Cruz, California 95064, USA; ^3^Department of Chemical and Materials Engineering, University of Alberta, Edmonton, Alberta, Canada T6G 1H9; ^4^Department of Physics, University of California, 1156 High Street, Santa Cruz, California 95064, USA; ^5^X-Ray Absorption Group, National Synchrotron Radiation Research Center, Hsinchu 30076, Taiwan; ^6^Institute of Super-Microstructure and Ultrafast Process in Advanced Materials, School of Physics and Electronics, Central South University, Changsha 410083, China; ^7^Key Laboratory of Materials Processing and Mold (Zhengzhou University), Ministry of Education, Zhengzhou 450002, China

## Abstract

Rational design of single-metal atom sites in carbon substrates by a flexible strategy is highly desired for the preparation of high-performance catalysts for metal-air batteries. In this study, biomass hydrogel reactors are utilized as structural templates to prepare carbon aerogels embedded with single iron atoms by controlled pyrolysis. The tortuous and interlaced hydrogel chains lead to the formation of abundant nanowrinkles in the porous carbon aerogels, and single iron atoms are dispersed and stabilized within the defective carbon skeletons. X-ray absorption spectroscopy measurements indicate that the iron centers are mostly involved in the coordination structure of FeN_4_, with a minor fraction (ca. 1/5) in the form of FeN_3_C. First-principles calculations show that the FeN_*x*_ sites in the Stone-Wales configurations induced by the nanowrinkles of the hierarchically porous carbon aerogels show a much lower free energy than the normal counterparts. The resulting iron and nitrogen-codoped carbon aerogels exhibit excellent and reversible oxygen electrocatalytic activity, and can be used as bifunctional cathode catalysts in rechargeable Zn-air batteries, with a performance even better than that based on commercial Pt/C and RuO_2_ catalysts. Results from this study highlight the significance of structural distortions of the metal sites in carbon matrices in the design and engineering of highly active single-atom catalysts.

## 1. Introduction

Climate change and environmental pollution have motivated the development of sustainable, clean energy technologies, of which rechargeable metal-air batteries have drawn tremendous attention owing to their high energy density and minimal impacts on the environment [1–4]. The overall efficiency of the charge-discharge process of metal-air batteries is determined by two major reactions, oxygen reduction reaction (ORR) and oxygen evolution reaction (OER). Although platinum group metal (PGM) materials, such as Pt/C, RuO_2_, and Ir/C, possess excellent catalytic activity for either ORR or OER, none of these noble metal catalysts displays a satisfactory performance for both [5], and their scarcity and high costs greatly hinder their practical applications [6, 7]. Therefore, development of bifunctional catalysts with a low cost and high activity is of both fundamental and technological significance, but remains a great challenge.

Recent studies have demonstrated that PGM-free nanocomposites based on carbon materials, such as heteroatom- (including nonmetal and metal atoms) doped porous carbon, are promising bifunctional oxygen catalysts [8–10]. In fact, transition metal-doped carbon catalysts have been widely investigated due to the unique chemical properties caused by their adjustable 3D electronic orbitals. In particular, transition metal-based single-atom catalysts display overwhelming superiority as compared to their nanoparticle and nanocluster counterparts [11–15]. For instance, single-site dispersion of FeN_*x*_ species in a two-dimensional nitrogen-doped porous carbon layer has been found to exhibit a remarkable catalytic activity towards both ORR and OER in alkaline media [1], where a range of catalytic active sites has been proposed, such as CoN_2_C_2_, FeN_3_C, FeN_4_, and FeN_4_O [16–18]. However, the effects of structural distortion induced by the single-metal sites on the catalytic activity have long been ignored, although such structural defects are common in pyrolytic carbon.

Herein, biomass hydrogels (i.e., chitosan, gelatin, and agar), which have long been known for their diverse applications and economic advantages [19, 20], were prepared and used as unique precursors, templates, and reactors to produce three-dimensional, nanowrinkled carbon aerogels embedded with FeN_*x*_ single sites [21–23]. Due to the abundant functional groups on the hydrogel chains, defective single-metal sites were dispersed and stabilized within the nanowrinkled, porous carbon aerogels. First-principles calculations showed that the FeN_*x*_ sites in the Stone-Wales configurations induced by the carbon nanowrinkles displayed a much lower free energy for oxygen electrocatalysis than the normal counterparts. Electrochemical measurements exhibited apparent and reversible oxygen electrocatalytic performance towards both ORR and OER. When the nanowrinkled carbon aerogels were used as the air-cathode of a zinc-air battery, the battery displayed a higher open-circuit voltage and higher energy density, as well as better cycling stability than that with commercial Pt/C-RuO_2_ catalysts.

## 2. Results and Discussion

### 2.1. Synthesis and Characterization

**Figure 1:**
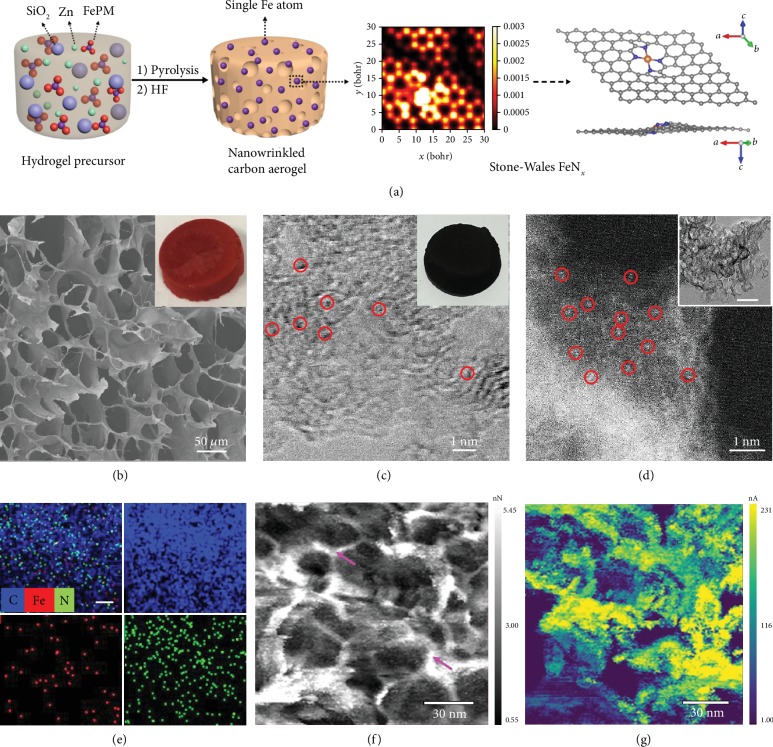
Synthesis and morphological characterization of the NCA_C-Zn_/Fe carbon aerogel. (a) Schematic representation of the synthesis of the NCA_C-Zn_/Fe carbon aerogel. (b) SEM image of the freeze-dried CS_Si-Zn_/FePM hydrogel. Inset is a digital photo of the sample. (c) Bright-field STEM image of the NCA_C-Zn_/Fe aerogel. Red circles indicate single Fe atoms. Inset is a digital photo of the sample. (d) Dark-field STEM image of the NCA_C-Zn_/Fe aerogel. Red circles indicate single Fe atoms. Inset is a TEM image of the NCA_C-Zn_/Fe aerogel, and the scale bar is 30 nm. (e) Elemental maps of NCA_C-Zn_/Fe aerogels. The scale bar is 10 nm. AFM images of NCA_C-Zn_/Fe aerogels: (f) adhesion force image and (g) current flow image.

In this study, flexible biomass hydrogels were synthesized in a facile process and employed as 3D templates to prepare carbon aerogels embedded with single-metal atoms (Figures 1(a) and [Supplementary-material supplementary-material-1]). In order to achieve atomic dispersion, the hydrogel networks were modified by two strategies to minimize metal aggregation. The first is “headstream fixation,” which means immobilization of metal atoms into the hydrogel reactor by complexation agents (e.g., phenanthroline (PM)); and the other, “roadblocks,” is based on rigid templates, such as SiO_2_ nanoparticles and Zn atoms. Experimentally, a variety of hydrogel/hydrosol networks, i.e., CS_Si-Zn_/FePM, CS_Si_/FePM, CS_Si_/Fe, and CS_Si_, were prepared by using chitosan (CS) as the structural scaffold, along with a select combination of other precursors, such as SiO_2_ nanoparticles (Si), FePM, and Zn salt (details in Materials and Methods). The morphological details were first investigated by scanning electron microscopy (SEM) measurements. From [Supplementary-material supplementary-material-1], freeze-dried CS_Si_/FePM, CS_Si_/Fe, and CS_si_ hydrosols can be seen to consist of uneven microcavities. However, as shown in Figure 1(b), the microcavities of the CS_Si-Zn_/FePM hydrogel shows a more uniform size of ca. 50 *μ*m, forming a 3D, continuous framework composed of intertwining CS-Zn chains. This suggests that Zn ions can induce the hydrogelation of CS hydrosol to form much more uniform 3D intertwining networks, which further facilitates the generation of nanowrinkles [24]. Circular dichroism (CD) and UV-vis absorption measurements were then carried out to monitor the structural evolution from CS_Si_ sol to CS_Si-Zn_/FePM hydrogel. As depicted in [Supplementary-material supplementary-material-1], the incorporation of both FePM and Zn^2+^ ions into chitosan led to marked conformational changes of the CS chains. In UV-vis absorption measurements ([Supplementary-material supplementary-material-1]), two new peaks can be seen to emerge at ca. 226 nm and 268 nm, due to the strong complexation interaction between Fe and PM [25].

The freeze-dried CS_Si-Zn_/FePM hydrogel was then used as a 3D reactor to synthesize metal-doped carbon aerogels by controlled pyrolysis, which was then subject to HF etching to remove the SiO_2_ templates (Figure 1(a)), producing NCA_C-Zn_/Fe (Figure 1(c) inset). Control samples of NCA_C_/Fe, CA_C_/Fe, and CA_C_ were prepared in a similar fashion (details in Materials and Methods). From the transmission electron microscopy (TEM) images in [Supplementary-material supplementary-material-1], one can see that the NCA_C-Zn_/Fe sample displays a highly porous, nanowrinkled structure with rich mesopores of ca. 10 nm in diameter. In both bright-field (Figure 1(c)) and dark-field (Figure 1(d)) scanning transmission electron microscopy (STEM) images, one can clearly see the formation of single-metal atoms (red circles) embedded into the porous carbon matrix. The corresponding elemental maps clearly show that the C, N, and Fe elements are homogeneously distributed across the aerogel (Figure 1(e)). These results indicate successful construction of N-doped carbon aerogels embedded with isolated Fe atoms by using biomass hydrogels as the reactors.

In order to examine the mechanical and electrical properties of the obtained porous carbon, Fast Force Mapping (FFM) measurements were then carried out. The data presented in Figures 1(f)–1(g) and [Supplementary-material supplementary-material-1] exhibit a ca. 10 nm variation in the mechanical and electrical properties of the porous carbon, confirming the formation of nanowrinkled carbon. Domains dictated by round features in topography ([Supplementary-material supplementary-material-1]) are outlined by prominent changes in max force ([Supplementary-material supplementary-material-1]) and an increase in the adhesion force (Figure 1(f)). Notably, the adhesion force, which represents the bulk modulus or stiffness of the sample, indicates that these round regions are stiffer in the center and softer around the edges. Typically, sp^2^-hybridized carbon exhibits hydrophobic characteristics, whereas defective carbons are more hydrophilic [26, 27]. With an AFM tip that consists of a hydrophilic silicon oxide layer, a high adhesion force corresponds to a hydrophilic domain. This implies that the metal centers are most likely situated within the high adhesion force areas. Interestingly, from Figures 1(f)to 1(g), one can see that the soft nodes correspond to high electrical conductance. Taken together, these results suggest that the metal sites are mostly located in the high adhesion and high conductivity areas of the porous carbon aerogel [28, 29]. Both features are conducive to oxygen electrocatalysis.

**Figure 2:**
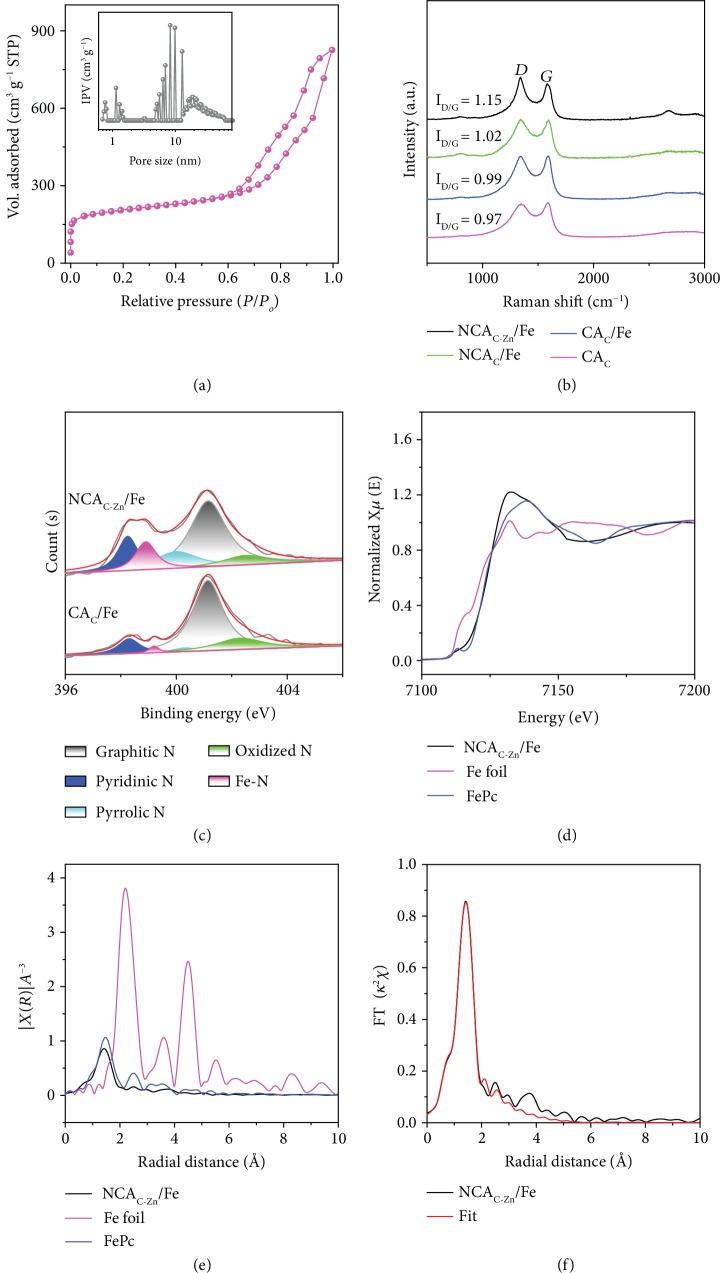
Structural characterization of the NCA_C-Zn_/Fe carbon aerogel. (a) N_2_ adsorption-desorption isotherm of NCA_C-Zn_/Fe. Inset is the corresponding pore size distribution. (b) Raman spectra of CA_C_, CA_C_/Fe, NCA_C_/Fe, and NCA_C-Zn_/Fe. (c) XPS spectra of the N 1s electrons in CA_C_/Fe and NCA_C-Zn_/Fe. (d) K-edge XANES of NCA_C-Zn_/Fe, FePc, and Fe foil. (e) K-edge EXAFS of NCA_C-Zn_/Fe, FePc, and Fe foil. (f) The corresponding EXAFS fitting curves for the NCA_LR_/Fe sample.

The porosity of the obtained samples was then quantitatively evaluated by N_2_ adsorption-desorption measurements. The carbon aerogels obtained above all show a Type IV isotherm (Figures 2(a) and [Supplementary-material supplementary-material-1]), which suggests the formation of a complex porous network containing a myriad of mesopores with an average size of ca. 10 nm, in line with the diameter of the SiO_2_ nanoparticle [30]. From the isotherms, the specific surface area of NCA_C-Zn_/Fe was estimated to be 609 m^2^ g^−1^, with a microporous surface area of 111 m^2^ g^−1^, which is the highest among the series, a condition favorable for the formation of abundant active sites ([Supplementary-material supplementary-material-1] and [Supplementary-material supplementary-material-1]). The corresponding X-ray powder diffraction (XRD) patterns are shown in [Supplementary-material supplementary-material-1], where only one broad peak at ca. 25° can be observed, due to the (002) diffraction of graphitic carbon [31]. This carbon diffraction became gradually sharpened from CS_C_ to NCA_C-Zn_/Fe, indicating an increasing degree of graphitization. Importantly, the fact that no other diffraction features were observed suggests the absence of metal (oxide) nanoparticles. In Raman measurements, the *I*_*D*_/*I*_*G*_ ratio of NCA_C-Zn_/Fe was estimated to be ca. 1.15, much higher than those of the control samples (Figure 2(b)), signifying the generation of rich defects which may be conducive to the formation of metal active sites [31].

The elemental compositions of the obtained carbon samples were then quantitatively assessed by inductively coupled plasma atomic emission spectroscopy (ICP-OES) and energy-dispersive X-ray spectroscopy (EDS) measurements. Results from ICP-OES analysis showed that the Fe content in the carbon aerogel was about 0.22 wt% for CA_C_/Fe, 0.61 wt% for NCA_C_/Fe, and 0.72 wt% for NCA_C-Zn_/Fe, in good accordance with the EDS results ([Supplementary-material supplementary-material-1], Tables [Supplementary-material supplementary-material-1]). The increased metal content suggests the important roles of PM (chelation) and Zn^2+^ ions (porogen and gel initiator) into fixing Fe centers in the carbon matrix. Further analysis was conducted with X-ray photoelectron spectroscopy (XPS) measurements. First of all, no Fe−O peak can be resolved in the high-resolution scan of the O 1s electrons ([Supplementary-material supplementary-material-1]), suggesting that Fe atoms are most likely coordinated to other atomic sites such as N and C; and from the XPS spectra of the N 1s electrons of the series of samples (Figures 2(c), [Supplementary-material supplementary-material-1]), one can see that the successive introduction of PM and Zn^2+^ into the precursors increased the N doping from 2.25 at% to 3.60 at% in the carbon matrix (Tables [Supplementary-material supplementary-material-1]), and the pyridinic N fraction was the highest in the NCA_C_/Fe (0.52 at%) and NCA_C-Zn_/Fe (0.51 at%) samples. In addition, as compared to CS_C_/Fe, the much stronger Fe-N peak (0.44 at% vs. 0.04 at%) in the NCA_C-Zn_/Fe sample suggests the generation of more abundant FeN_*x*_ moieties in the carbon aerogels.

The structural configuration of the FeN_*x*_ functional moiety was then examined by X-ray absorption spectroscopy (XAS) measurements. From Figure 2(d), one can see that the Fe K-edge X-ray absorption near-edge spectrum (XANES) of NCA_C-Zn_/Fe is very similar to that of FePc but markedly different from that of an Fe foil, suggesting a comparable oxidation state (+2) of the Fe centers in NCA_C-Zn_/Fe and FePc. In the extended X-ray absorption fine structure (EXAFS) spectrum of the Fe foil, the Fe-Fe peak is well-defined at 2.21 Å (Figure 2(e)); however, this peak is absent in NCA_C-Zn_/Fe, consistent with the atomic dispersion of Fe in the NCA_C-Zn_/Fe sample (Figure 1). In fact, both the NCA_C-Zn_/Fe and FePc samples display only a single major peak at 1.41 Å, which can be assigned to the Fe-N bond. Furthermore, the first shell of NCA_C-Zn_/Fe is well fitted with 3.8 N and 0.2 C with the same bond length of 1.94 Å (Figures 2(f) and [Supplementary-material supplementary-material-1]). Taken together, these results suggest that the Fe centers in NCA_C-Zn_/Fe were mostly involved in the coordination structure of FeN_4_, with a minor fraction (ca. 1/5) in the form of FeN_3_C.

### 2.2. Theoretical Investigation of the ORR Using the NCA_C-Zn_/Fe Catalyst

**Figure 3:**
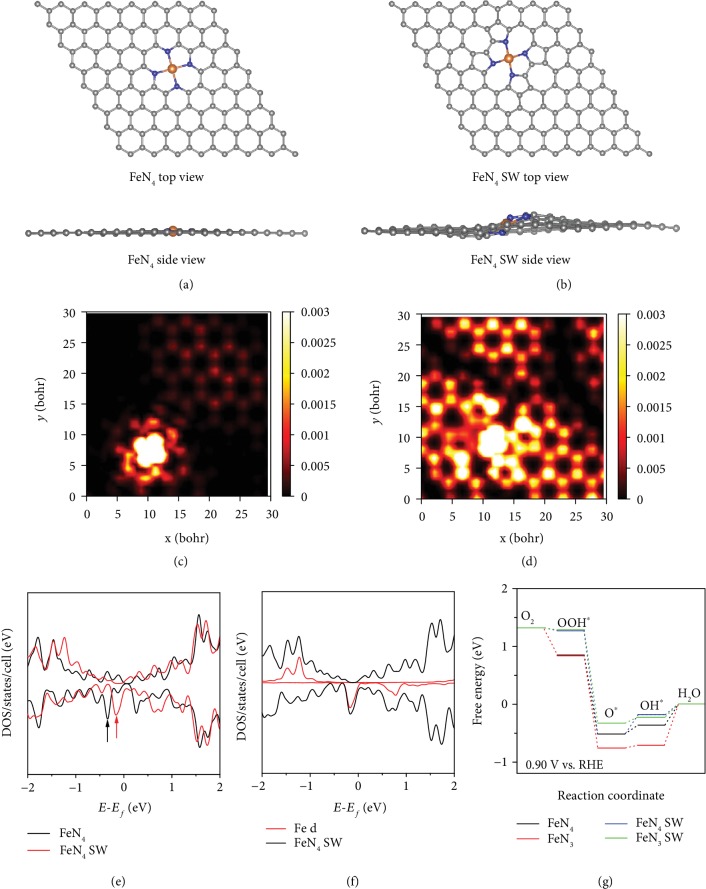
Atomic model and DOS of metal sites; Gibbs free energy diagrams of ORR. (a, c) Side/top view and simulated STM image (at a bias of -1.0 V) of normal FeN_4_ doped graphene sheets. (b, d) Side/top view and simulated STM image (at a bias of -1.0 V) of Stone-Wales FeN_4_- (FeN_4_ SW-) doped graphene sheets. (e) Density of state (DOS) of normal FeN_4_ and FeN_4_ SW-doped graphene sheets. (f) DOS of FeN_4_ SW and Fe 3d. (g) Free energy diagrams of ORR processes on normal FeN_4_, normal FeN_3_, FeN_4_ SW, and FeN_3_ SW at the applied potential of +0.9 V vs. RHE.

First-principles calculations were then carried out to shed light on the contributions of FeN_4_ and FeN_3_C moieties to the electrocatalytic activity. It is likely that the interlaced 3D structure of the hydrogel networks and the tortuous CS chains can lead to the formation of abundant wrinkles in the obtained porous carbon aerogels. Therefore, the electrocatalytic activities of the Stone-Wales- (SW-) defect FeN_4_ (FeN_4_ SW) and FeN_3_C (FeN_3_C SW) moieties, which can be formed by the nanowrinkles of carbon matrices, are examined by theoretical calculations. Figures 3(a)–3(b) and [Supplementary-material supplementary-material-1] show the side view and top view of the atomic models of the four kinds of Fe-N centers. From the atomic models, one can see that the normal FeN_4_ and normal FeN_3_C moieties exhibit a planar structure in the carbon matrices, while FeN_4_ SW and FeN_3_C SW show a distorted nonplanar structure. The simulated scanning tunneling microscopic (STM) images of the FeN_4_ and FeN_4_ SW moieties are presented in Figures 3(c)–3(d) and [Supplementary-material supplementary-material-1]. As compared to normal FeN_4_, the SW defects cause significant redistribution of electron densities of FeN_4_ and adjacent carbon atoms.  Figure 3(e) displays the total density of states (DOS) of normal and SW Fe-N centers. According to Figure 3(f), for the FeN_4_ SW on a graphene sheet, the Fe atom makes the largest contributions to the DOS near the Fermi level (red peak), which is similar to that (black line) of normal FeN_4_. Apparently, the marked state of FeN_4_ SW (highlighted by arrows in Figure 3(e)) is much closer to the Fermi level than that of normal FeN_4_, indicating a higher probability of donating electrons and reducing oxygen.

To evaluate the ORR activity of these Fe-N metal centers, the reaction free energy is calculated at the applied potential of +0.9 V vs. RHE and plotted in Figure 3(g). One can see that the first two electron-transfer steps are exothermic and the last two endothemic, with the rate determining step (RDS) most likely the fourth electron-transfer step of water formation and desorption. In comparison with normal FeN_4_ and FeN_3_, both FeN_4_ SW and FeN_3_ SW show much lower endothermic energies (0.179 eV and 0.228 eV), implying a lower reaction overpotential. These results suggest that the nanowrinkles can enhance the electrocatalytic activity of Fe-N centers on the carbon matrices by forming SW defects, as manifested below in electrochemical tests.

### 2.3. Electrocatalytic Activity towards ORR

**Figure 4:**
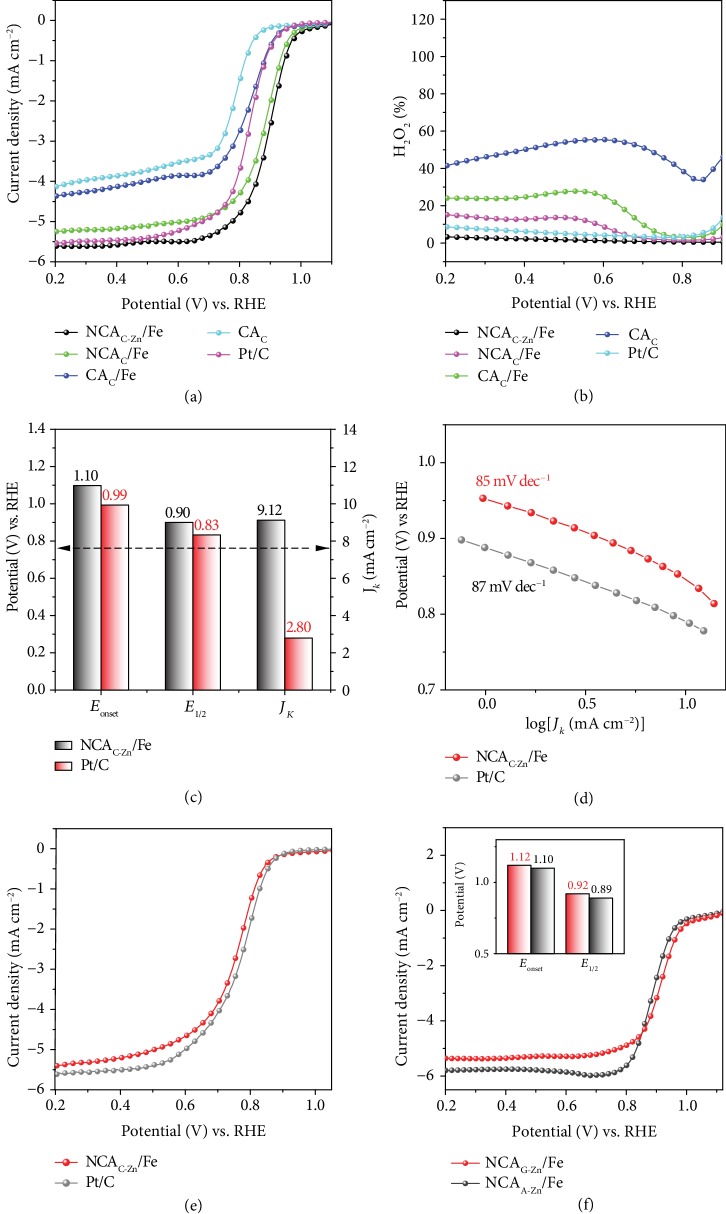
ORR performance. (a) ORR polarization curves of CA_C_, CA_C_/Fe, NCA_C_/Fe, and NCA_C-Zn_/Fe, as well as the Pt/C at 1600 rpm in 0.1 M KOH at the potential sweep rate of 5 mV s^−1^. (b) H_2_O_2_ yield of CA_C_, CA_C_/Fe, NCA_C_/Fe, NCA_C-Zn_/Fe, and Pt/C. (c) *E*_onset_, *E*_1/2_, and *J*_*k*_ (at +0.85 V) of the NCA_C-Zn_/Fe carbon aerogels and Pt/C catalyst. (d) Tafel plots of NCA_C-Zn_/Fe and Pt/C. (e) ORR performance in acidic media. ORR polarization curves of the NCA_C-Zn_/Fe and the Pt/C at 1600 rpm on RDE in 0.1 M HClO_4_ at the potential sweep rate of 5 mV s^−1^. (f) ORR activity of aerogels derived from agar and gelatin hydrogels. ORR polarization curves of NCA_A-Zn_/Fe and NCA_G-Zn_/Fe as well as Pt/C at 1600 rpm on RDE in 0.1 M KOH at the potential sweep rate of 5 mV s^−1^.

The electrocatalytic activity of the nanowrinkled carbon aerogels obtained above was then investigated in 0.1 M KOH. First, electrical impedance spectroscopy (EIS) analysis was carried out to investigate the electron-transfer kinetics. For the NCA_C-Zn_/Fe catalyst, the small diameter at high frequency and the steep tail at low frequency suggest excellent channels for both mass transfer and charge transfer. Such a low impedance is anticipated to facilitate ORR electrocatalysis ([Supplementary-material supplementary-material-1]). Figures 4(a) and 4(b) show the ORR polarization curves and H_2_O_2_ yields of the carbon aerogels, in comparison to commercial Pt/C (20 wt%). As a metal-free catalyst, the CA_C_ sample shows a rather apparent electrocatalytic activity with an onset potential (*E*_onset_) of +0.94 V vs. RHE and a half-wave potential (*E*_1/2_) of +0.79 V, much more positive than those of other carbon catalysts reported in recent literature [15, 32, 33]. This suggests that biomass alone may be exploited as a carbon source to fabricate metal-free ORR electrocatalysts. Notably, doping of the FePM complex into the CS_Si_ leads to a marked enhancement of the catalytic performance with *E*_onset_ = +1.10 V and *E*_1/2_ = +0.90 V (NCA_C-Zn_/Fe), which is even better than those of commercial Pt/C (+0.99 V and+ 0.83 V) [34]. Likewise, the NCA_C-Zn_/Fe single-atom catalyst shows the lowest average H_2_O_2_ yield (1.45%) within the potential range of +0.2 V to +0.9 V, signifying a high-efficiency 4*e*^−^ reduction pathway (Figures [Supplementary-material supplementary-material-1]). From the Koutecky-Levich plots, the kinetic current density (*J*_*k*_) at +0.85 V was estimated to be 9.12 mA cm^−2^, about 3.3 times that of Pt/C (2.80 mA cm^−2^, Figure 4(c)). Both NCA_C-Zn_/Fe and Pt/C show a low Tafel slope (85 vs. 87 mV dec^−1^) in the high potential range, illustrating an efficient kinetic process of ORR on these two catalysts (Figure 4(d)). Besides, in contrast with Pt/C, the NCA_C-Zn_/Fe exhibits remarkable durability and methanol tolerance ([Supplementary-material supplementary-material-1]). In addition, the *E*_1/2_ and diffusion-limited current density of NCA_C-Zn_/Fe were comparable to those of Pt/C in acidic media (0.1 M HClO_4_), suggesting the high ORR activity of the single iron atom catalysts even at low pH (Figure 4(e)).

To distinguish the contributions of the Fe center and adjacent nonmetal atoms to the electrocatalytic activity, electrochemical measurements were then carried out with the addition of SCN^−^ as the poisoning species. One can see that upon the addition of 10 mM SCN^−^ into 0.1 M KOH, the *E*_1/2_ of NCA_C-Zn_/Fe exhibited a negative shift of 20 mV ([Supplementary-material supplementary-material-1]). The relatively mild performance deterioration suggests that in addition to the Fe sites, adjacent nonmetal atoms also play a critical role in driving the catalytic reaction. This is actually in good agreement with the formation of FeN_*x*_ SW moieties in the carbon skeletons, where structural distortion leads to the activation of adjacent C atoms (Figures 3 and [Supplementary-material supplementary-material-1]) [8].

Notably, other biomass hydrogels, such as gelatin and agar, can also be used as templates to fabricate nanowrinkled carbon aerogels embedded with single-metal atoms in a similar fashion. The resulting catalysts, NCA_G-Zn_/Fe and NCA_A-Zn_/Fe, both displayed excellent catalytic activities towards ORR in alkaline media, with an *E*_1/2_ of +0.92 and +0.89 V and an *E*_onset_ of +1.12 and 1.10 V, respectively (Figure 4(f) and inset). These results highlight the universality of the synthetic strategy in the preparation of high-performance ORR electrocatalysts ([Supplementary-material supplementary-material-1]).

### 2.4. Electrocatalytic Activity towards OER and Zinc-Air Battery Performance

**Figure 5:**
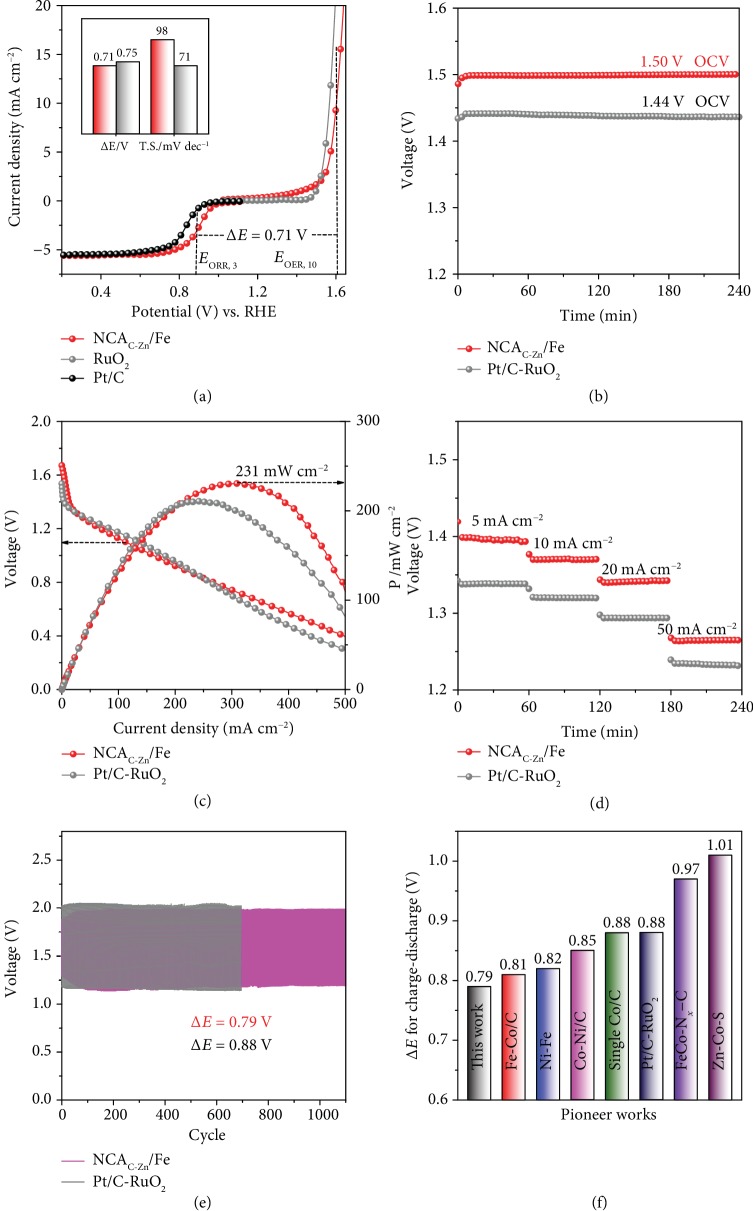
Zn-air performance tests. (a) Polarization curves for OER and ORR by NCA_C-Zn_/Fe and Pt/C-RuO_2_. Inset shows the corresponding Δ*E* between *E*_*j*=3_ and *E*_*j*=10_ and the Tafel slope of OER. (b) OCV and (c) power densities of Zn-air batteries assembled by NCA_C-Zn_/Fe and Pt/C-RuO_2_. (d) Discharge tests at various current densities (5, 10, 20, and 50 mA cm^−2^) of NCA_C-Zn_/Fe and Pt/C-RuO_2_. (e) Charge-discharge curves of Zn-air batteries assembled by NCA_C-Zn_/Fe and Pt/C-RuO_2_ at 10 mA cm^−2^ for 1100 cycles (400 s per cycle). (f) Comparison of charge-discharge voltage gap for NCA_C-Zn_/Fe with leading results reported in recent literature [5, 11, 37–40].

The electrocatalytic activity of the NCA_C-Zn_/Fe aerogels towards OER was then examined and compared with commercial RuO_2_ in 1 M KOH with iR correction. From Figure 5(a), one can see that for NCA_C-Zn_/Fe, an overpotential (*η*_10_) of +370 mV was needed to achieve the current density of 10 mA cm^−2^, a performance comparable to that of commercial RuO_2_ (*η*_10_ = +340 mV). The NCA_C-Zn_/Fe also exhibits a Tafel slope of 98 mV dec^−2^, close to that of RuO_2_ (71 mV dec^−2^), signifying a favorable OER kinetic ([Supplementary-material supplementary-material-1]). Thanks to the excellent electrocatalytic performance towards both ORR and OER, the NCA_C-Zn_/Fe SACs show a low potential difference (Δ*E*) of only 0.71 V between the OER potential at 10 mA cm^−2^ (*E*_OER,10_) and the ORR potential at 3 mA cm^−2^ (*E*_ORR,3_), much smaller than those of bifunctional M−N−C catalysts reported recently in the literature [11, 35, 36].

With such a remarkable bifunctional performance, the NCA_C-Zn_/Fe aerogels were tested as the air-cathode for a Zn-air battery, in comparison with those using a commercial Pt/C-RuO_2_ mixture (mass ratio 1 : 1), along with a Zn plate as the anode. From Figures 5(b)to 5(c), the NCA_C-Zn_/Fe-Zn-air battery can be seen to show an open-circuit voltage (OCV) of 1.50 V and a maximum power density of 231 mW cm^−2^, about 6 mV and 20 mW cm^−2^ higher than those of the Pt/C-RuO_2_ counterpart. Figure 5(d) shows the corresponding constant current discharge tests at various current densities (5, 10, 20, and 50 mA cm^−2^) of the two batteries. One can see that the NCA_C-Zn_/Fe-Zn battery exhibited a much higher discharge voltage within a wide range of current densities (5 to 50 mA cm^−2^). At the constant current density of 10 mA cm^−2^, the NCA_C-Zn_/Fe-Zn battery displayed a stable and optimal potential of 1.36 V for 41 h. By normalizing the energy output to the weight of dissipated Zn, the calculated specific capacity and energy density were estimated to be 780 mAh g^−1^ and 956 Wh kg^−1^, respectively, markedly higher than those of Pt/C-RuO_2_ ([Supplementary-material supplementary-material-1]). Also, the small charge-discharge voltage gap of the NCA_C-Zn_/Fe-Zn battery in Figure 5(e) indicates excellent rechargeability ([Supplementary-material supplementary-material-1]). Impressively, the battery also delivers a stable potential plateau in the charge-discharge test at the constant current density of 10 mA cm^−2^ during prolonged operation. After 1100 continuous charge-discharge cycles (400 s for each cycle), the NCA_C-Zn_/Fe-Zn battery still afforded a high round-trip efficiency of 59% and a narrow discharge-recharge voltage gap of 0.79 V, much better than those of Pt/C-RuO_2_ and other leading oxygen electrocatalysts reported in recent literature (Figure 5(f)) [5, 11, 37–40]. Taken together, these results demonstrate that the NCA_C-Zn_/Fe aerogels derived from biomass hydrogels can be used as high-performance bifunctional oxygen electrodes for Zn-air batteries, thanks to its high open-circuit voltage, large power density, and superb durability.

## 3. Conclusion

In this study, a facile, scalable strategy was developed for the preparation of nanowrinkled carbon aerogels embedded with FeN_*x*_ active sites by utilizing biomass hydrogels as the precursors and reactors. The resulting nanowrinkled carbon aerogels (NCA_C-Zn_/Fe) showed an excellent and reversible ORR/OER electrocatalytic performance with a low voltage gap of only 0.71 V for oxygen electrocatalysis. With the obtained carbon aerogels as the (air) cathode catalysts of a Zn-air battery, the battery exhibited a higher open-circuit voltage, greater power density, and superior durability than that based on a mixture of commercial Pt/C-RuO_2_ catalysts. First-principles calculation showed that FeN_*x*_ sites in Stone-Wales defect formed by the carbon nanowrinkles were most likely responsible for the excellent electrocatalytic activity. Results from the present study suggest that creating structural distortion of metal sites in carbon matrices can be exploited as an effective strategy for the design and engineering of advanced electrocatalysts based on atomically dispersed metal centers.

## 4. Materials and Methods

### 4.1. Reagents

Potassium hydroxide (KOH), iron(II) chloride tetrahydrate (FeCl_2_·4H_2_O), SiO_2_ nanoparticles (15 nm), zinc(II) acetate (Zn(OAc)_2_), potassium thiocyanate (KSCN), gelatin, and agar were purchased from Aladdin Reagents (Shanghai, China). Perchloric acid (HClO_4_) and ammonium hydroxide (NH_4_OH) were purchased from Xiya Reagents (Chengdu, China). Chitosan and Nafion solution were purchased from Sigma-Aldrich (USA). Commercial Pt/C (20 wt%) and high-purity zinc plate (99.999%) were obtained from Johnson Matthey. Polytetrafluoroethylene (PTFE, 60 wt%, D-210C) was purchased from Japan DaJin. All other reagents were analytical grade, and ultrapure water (Mill-Q, 18.3 MΩ cm) was used throughout this study.

### 4.2. Instrumentation

TEM studies were carried out with a T20 FEI Tecnai G2 instrument. Scanning electron microscopy images were obtained with a Hitachi S-4800 field-emission scanning electron microscope. STEM with EDS studies were performed on a JEOL JEM-ARM200CF with aberration-corrected STEM. Topography, maximum force, adhesion force, and current flow were measured using the Fast Force Mapping technique with an Oxford Instruments Asylum Cypher S AFM housed in an Ar-gas-filled glove box. Raman spectra were acquired with a Renishaw inVia Raman microscope. CD spectra were recorded on Jasco J-815 CD spectrometer (Japan). UV-vis spectra were acquired with a Shimadzu UV-2450 Spectrophotometer (Japan). ICP-OES studies were performed on a SPECTROBLUE SOP instrument. XRD and XPS measurements were carried out on a D/MAX2550 X-Ray Power Diffractometer and a Thermo Fisher-VG Scientific ESCALAB 250Xi X-Ray Photoelectron Spectrometer, respectively. N_2_ adsorption-desorption isotherms were obtained with a Micromeritics ASAP 2020 Surface Area and Porosity Analyzer. Fe K-edge EXAFS measurements were performed at the Quick-EXAFS Beamline of the Taiwan Photon Source in transmission mode, and the results were analyzed by using the FeN_*x*_C_*y*_ structural model with the Athena program. An RST 5200F electrochemical workstation (Zhengzhou, China) was used to perform the voltammetric measurements. Rotating Disk Electrode (RDE, Pine Research Instrument) tests were carried out at the rotation rates of 400 to 1600 rpm.

### 4.3. Synthesis of Catalysts

In a typical reaction, 60 mg of chitosan (CS), 40 mg of SiO_2_ nanoparticles (15 nm), and 2.56 mL of acetic acid (1%) were placed in screw-cap vial under magnetic stirring for 90 min, into which was added 90 *μ*L of NH_3_H_2_O (20 wt%) to adjust the solution pH to 7.0. 200 *μ*L of 0.2 M Fe-phenanthrolene (Fe(PM)_3_^2+^) and 80 *μ*L of 1 M zinc acetate (Zn(OAc)_2_) were then added into the above solution under stirring. Sonication treatment for 6 min yielded a hydrogel, which was denoted as CS_Si-Zn_/FePM (“Si,” “Zn,” and “FePM” stand for SiO_2_ nanoparticles, Zn^2+^ ions, and Fe(PM)_3_^2+^, respectively). The CS_Si-Zn_/FePM hydrogel obtained above was freeze-dried and then heated to 900°C at the heating rate of 5°C min^−1^ in an Ar atmosphere (containing 3% H_2_). After heating at 900°C for 3 h, the sample was cooled down to room temperature and subjected to HF etching to remove SiO_2_ nanoparticles, affording an Fe-N-codoped carbon aerogel, which was referred to as NCA_C-Zn_/Fe.

Three control samples were prepared in the same fashion except that only one or two of the starting materials (SiO_2_ nanoparticles, Zn^2+^ ions, and Fe(PM)_3_^2+^) were used to prepare the biomass hydrogel precursors (i.e., CS_Si_, CS_Si_/Fe, and CS_Si_/FePM). The corresponding aerogels were denoted as CA_C_, CA_C_/Fe, and NCA_C_/Fe, respectively.

### 4.4. Electrochemistry

Electrochemical tests were carried out in a three-electrode electrochemical cell with a graphite rod as the counter electrode and an Ag/AgCl (saturated KCl) electrode as the reference electrode. The Ag/AgCl reference electrode was calibrated against a reversible hydrogen electrode (RHE) and all potentials in the present study were referenced to this RHE. To prepare a catalyst ink, 3 mg of the catalysts obtained above was dispersed in a 475 *μ*L mixed solvent of H_2_O and ethanol (*v* : *v* = 1 : 1) and 25 *μ*L of a Nafion solution (5%) under sonication for 1 h to form a homogeneous dispersion (6 mg mL^−1^). For ORR tests, the catalyst ink was loaded onto a cleaned glassy carbon electrode at the catalyst loading of 250 *μ*g cm^−2^ for cyclic voltammetry and 400 *μ*g cm^−2^ for RDE and RRDE measurements in 0.1 M KOH, respectively. For OER tests, the catalyst was loaded onto a carbon paper at the mass loading of 1.0 mg cm^−2^ in 1.0 M KOH.

In RDE measurements the disk current density (*J*) is defined by the Koutecky-Levich (K-L) equation:

(1) $$\begin{array}{c} \frac{1}{J}=\frac{1}{J_{L}}+\frac{1}{J_{K}}=\frac{1}{B\omega^{1/2}}+\frac{1}{J_{K}},\\ B=0.2nFC_{0}{\left(D_{0}\right)}^{2/3}\upsilon^{-1/6}, \end{array}$$

where *J*_*L*_ is the limiting current density, *J*_*K*_ is the kinetic current density, *ω* is the rotation rate, *n* is electron transfer number, *F* is the Faraday constant (96,485 C mol^−1^), *C*_0_ is the O_2_ concentration in the electrolyte solution (1.2 × 10^−6^ mol cm^−3^), and *ν* is the kinematic viscosity of the electrolyte (0.01 cm^2^ s^−1^ for 0.1 M KOH). *J*_*K*_ can be determined from the intercept of the K-L plot (*J*^−1^ vs. *ω*^−1/2^).

In RRDE measurements, the H_2_O_2_ yield and electron transfer number (*n*) can be calculated by equation (2).

(2) $$\begin{array}{c} \%{\text{H}}_{2}{\text{O}}_{2}=200\times \frac{I_{r}/N}{I_{d}+\left(I_{r}/N\right)},\\ n=4\times \frac{I_{d}}{I_{d}+\left(I_{r}/N\right)}, \end{array}$$

where *I*_*r*_ and *I*_*d*_ are the ring current and disk current, respectively, and *N* is the collection efficiency of the ring electrode (0.37).

### 4.5. Fabrication of Home-Made Zinc-Air Battery

To make a zinc-air battery, a 6 M KOH solution containing 0.2 M zinc acetate was used as the electrolyte. A zinc plate was used as the anode. The air cathode was composed of three layers, i.e., a gas diffusion layer, a Ni foam layer, and a catalyst layer. The Ni foam was subject to sonication treatment in 0.1 M HCl, H_2_O, and ethanol consecutively, and vacuum dried at 80°C for 3 h. The catalyst layer was prepared by mixing 60 mg NCA_C-Zn_/Fe catalyst (or a mixture of 30 mg Pt/C and 30 mg RuO_2_), 10 mg acetylene black (as a conductive agent), and 30 mg PTFE emulsion. The total thickness of the cathode was ca. 0.4 mm after compression with a manual tablet machine and vacuum dried at 80°C for 3 h.

### 4.6. DFT Calculations

DFT calculations were carried out by Quantum ESPRESSO which is an open-source planewave code [41]. A two-dimensional supercell was built based on an 8 × 8 unit cell (127-129 atoms in total). For avoiding the interactions between periodic images, the vacuum at the *z*-axis was set at 14 Å. The ultrasoft pseudopotential was adopted [42]. The kinetic and charge density cutoff were set at 40 and 200 Ry, respectively. The 2 × 2 × 1 Monkhorst-Pack K-point grids were sampled for the supercell. The total energy was converged to 10^−3^ eV for geometric relaxation. The Marzari-Vanderbilt smearing was adopted with a smearing of 0.01 Ry [43]. The electronic energy and force were converged to 10^−8^ Ry and 10^−4^ a.u., respectively. The phonon contribution to zero-point energy and entropy was calculated based on the density functional perturbation theory [44, 45]. STM calculations were carried out based on the Tersoff and Hamann approximation [46] as implemented in the open-source Quantum ESPRESSO package [41] at a bias of -1.0 or +1.0 V, as described in the literature [47].

## Supplementary Materials

Supplementary Materials Table S1: BET surface area of CA_C_, CA_C_/Fe, NCA_C_/Fe, and NCA_C-Zn_/Fe. Table S2: EDS results of CA_C_, CA_C_/Fe, NCA_C_/Fe, and NCA_C-Zn_/Fe. Table S3: Fe contents in NCA_C-Zn_/Fe and NCA_C_/Fe determined by ICP-OES measurements. Table S4: elemental analysis by XPS measurements. Table S5: assignments of N species for different samples. Figure S1: schematic illustration of the preparation of NCA_C-Zn_/Fe carbon aerogels. Figure S2: SEM images of CS_Si_, CS_Si_/Fe, and CS_Si_/FePM. Figure S3: (a) CD and (b) UV-vis spectra of the series of biomass-derived hydrogels. Figure S4: TEM image of the NCA_C-Zn_/Fe aerogel. Figure S5: AFM images of NCA_C-Zn_/Fe aerogels: (a) topography image, (b) max force image, (c) adhesion force image, and (d) current flow image. Figure S6: (a) N2 absorption-desorption isotherm and (b) pore size distribution of CA_C_, CA_C_/Fe, NCA_C_/Fe, and NCA_C-Zn_/Fe. Figure S7: XRD patterns of CA_C_, CA_C_/Fe, NCA_C_/Fe, and NCA_C-Zn_/Fe. Figure S8: EDS profiles of CA_C_, CA_C_/Fe, NCA_C_/Fe, and NCA_C-Zn_/Fe. The results are summarized in Table S2. Figure S9: (a) XPS of O 1s electrons of NCA_C-Zn_/Fe. XPS of the N 1s electrons of (b) NCA_C_/Fe and (c) CA_C_. Figure S10: EXAFS fitting curves for NCA_C-Zn_/Fe. Inset is the corresponding K-space profiles. Figure S11: top view of normal FeN_3_ (a) and FeN_3_ SW (b) moieties in graphene; side view of normal FeN_3_ (c) and FeN_3_ SW (d) moieties. Figure S12: simulated STM image (at a bias of 1.0 V) of (a) normal FeN_4_ (a) and (b) Stone-Wales FeN_4_- (FeN_4_ SW-) doped graphene sheets. Figure S13: EIS spectra of CA_C_, CA_C_/Fe, NCA_C_/Fe, and NCA_C-Zn_/Fe. Figure S14: RRDE polarization curves of CA_C_, CA_C_/Fe, NCA_C_/Fe, and NCA_C-Zn_/Fe, as well as Pt/C at 1600 rpm in 0.1 M KOH. Potential scan rate: 5 mV s^−1^. Figure S15: (a) electron transfer numbers of the biomass-derived carbon aerogels and Pt/C at different potentials; (b) average electron transfer numbers of CA_C_ (4), CA_C_/Fe (3), NCA_C_/Fe (2), NCA_C-Zn_/Fe (1), and Pt/C. Figure S16: durability tests of NCA_C-Zn_/Fe and Pt/C; scan rate: 50 mV s^−1^; medium: O_2_-saturated 0.1 M KOH. (f) CV curves of the NCA_C-Zn_/Fe and commercial Pt/C as ORR catalysts in the presence or absence of 1 M MeOH. Figure S17: ORR polarization curves of NCA_C-Zn_/Fe in 0.1 M KOH with or without 10 mM KSCN. Figure S18: OER Tafel plots of (a) NCA_C-Zn_/Fe and (b) RuO_2_. Figure S19: specific capacity and energy density of NCA_C-Zn_/Fe and Pt/C-RuO_2_. Figure S20: charge-discharge tests of NCA_C-Zn_/Fe and Pt/C-RuO_2_.
