# Supplementary material for: Nanowrinkled Carbon Aerogels Embedded with FeNx Sites as Effective Oxygen Electrodes for Rechargeable Zinc-Air Battery
Source: Research (Wash D C). 2019 Dec 20;2019:6813585. doi: 10.34133/2019/6813585 (PMC6944486; doi:10.34133/2019/6813585)
Supplement: Supplementary Materials — Table S1: BET surface area of CAC, CAC/Fe, NCAC/Fe, and NCAC-Zn/Fe. Table S2: EDS results of CAC, CAC/Fe, NCAC/Fe, and NCAC-Zn/Fe. Table S3: Fe contents in NCAC-Zn/Fe and NCAC/Fe determined by ICP-OES measurements. Table S4: elemental analysis by XPS measurements. Table S5: assignments of N species for different samples. Figure S1: schematic illustration of the preparation of NCAC-Zn/Fe carbon aerogels. Figure S2: SEM images of CSSi, CSSi/Fe, and CSSi/FePM. Figure S3: (a) CD and (b) UV-vis spectra of the series of biomass-derived hydrogels. Figure S4: TEM image of the NCAC-Zn/Fe aerogel. Figure S5: AFM images of NCAC-Zn/Fe aerogels: (a) topography image, (b) max force image, (c) adhesion force image, and (d) current flow image. Figure S6: (a) N2 absorption-desorption isotherm and (b) pore size distribution of CAC, CAC/Fe, NCAC/Fe, and NCAC-Zn/Fe. Figure S7: XRD patterns of CAC, CAC/Fe, NCAC/Fe, and NCAC-Zn/Fe. Figure S8: EDS profiles of CAC, CAC/Fe, NCAC/Fe, and NCAC-Zn/Fe. The results are summarized in Table S2. Figure S9: (a) XPS of O 1s electrons of NCAC-Zn/Fe. XPS of the N 1s electrons of (b) NCAC/Fe and (c) CAC. Figure S10: EXAFS fitting curves for NCAC-Zn/Fe. Inset is the corresponding K-space profiles. Figure S11: top view of normal FeN3 (a) and FeN3 SW (b) moieties in graphene; side view of normal FeN3 (c) and FeN3 SW (d) moieties. Figure S12: simulated STM image (at a bias of 1.0 V) of (a) normal FeN4 (a) and (b) Stone-Wales FeN4- (FeN4 SW-) doped graphene sheets. Figure S13: EIS spectra of CAC, CAC/Fe, NCAC/Fe, and NCAC-Zn/Fe. Figure S14: RRDE polarization curves of CAC, CAC/Fe, NCAC/Fe, and NCAC-Zn/Fe, as well as Pt/C at 1600 rpm in 0.1 M KOH. Potential scan rate: 5 mV s−1. Figure S15: (a) electron transfer numbers of the biomass-derived carbon aerogels and Pt/C at different potentials; (b) average electron transfer numbers of CAC (4), CAC/Fe (3), NCAC/Fe (2), NCAC-Zn/Fe (1), and Pt/C. Figure S16: durability tests of NCAC-Zn/Fe and Pt/C; scan rate: 50 mV [file 6813585.f1.pdf]

Supporting Information

Nanowrinkled Carbon Aerogels Embedded with FeN_x Sites as Effective Oxygen Electrodes for Rechargeable Zinc-Air Battery

Ting He,[1,2,†] Bingzhang Lu,[2,†] Yang Chen,[1] Yong Wang,[1] Yaqiang Zhang,[3] John L. Davenport,[4] Alan P. Chen,[4] Chih-Wen Pao,[5] Min Liu,[6] Zhifang Sun,[1] Alexander Stram,[4] Alexander Mordaunt,[4] Jairo Velasco Jr.,[4] Yuan Ping,[2] Yi Zhang,[1,7,*] Shaowei Chen[2,*]

[1] Hunan Provincial Key Laboratory of Chemical Power Sources, College of Chemistry and Chemical Engineering, Central South University, Changsha 410083 (China)

[2] Department of Chemistry and Biochemistry, University of California, 1156 High Street, Santa Cruz, California 95064 (United States)

[3] Department of Chemical and Materials Engineering, University of Alberta, Edmonton, Alberta T6G 1H9 (Canada)

[4] Department of Physics, University of California, 1156 High Street, Santa Cruz, California 95064 (United States)

[5] X-ray Absorption Group, National Synchrotron Radiation Research Center, Hsinchu 30076 (Taiwan)

[6] Institute of Super-Microstructure and Ultrafast Process in Advanced Materials, School of Physics and Electronics, Central South University, Changsha 410083 (China)

[7] Key Laboratory of Materials Processing and Mold (Zhengzhou University), Ministry of Education, Zhengzhou 450002 (China)

*Corespondence should be address to Yi Zhang: yzhangcsu@csu.edu.cn; Shaowei Chen: shaowei@ucsc.edu.

† These authors contributed equally to this work.

1 Tables

Table S1 BET surface area of CA_C, CA_C/Fe, NCA_C/Fe and $NCA_{C\text{-}Zn}$/Fe.

Sample	S_{BET} (m^2 g^{-1})	Pore volumes (cm^3 g^{-1})	S_{BET} (m^2 g^{-1})		
			micropore	mesopore	macropore
$NCA_{C\text{-}Zn}$/Fe	608.5	1.11	110.7	475.9	21.9
NCA_C/Fe	649.2	1.56	66.2	553.9	29.1
CA_C/Fe	732.7	2.06	59.4	652.0	21.3
CA_C	666.8	2.44	35.0	563.5	68.3

Table S2 EDS results of CA_C, CA_C/Fe, NCA_C/Fe and $NCA_{C\text{-}Zn}$/Fe.

Elemental content (at%)	$NCA_{C\text{-}Zn}$/Fe	NCA_C/Fe	CA_C/Fe	CA_C
C	93.70	92.10	94.10	91.90
N	3.70	4.70	1.30	5.50
O	2.50	3.10	4.50	2.60
Fe	0.10	0.10	0	0

Table S3 Fe contents in $NCA_{C\text{-}Zn}$/Fe and NCA_C/Fe determined by ICP-OES measurements

Elements	$NCA_{C\text{-}Zn}$/Fe	NCA_C/Fe	CA_C/Fe	CA_C
Fe (wt%)	0.72	0.61	0.22	0

Table S4 Elemental analysis by XPS measurements.

Elemental content (at%)	$NCA_{C\text{-}Zn}$/Fe	NCA_C/Fe	CA/Fe	CA
C 1s	91.53	92.92	94.8	94.57
O 1s	4.48	3.55	2.74	2.26
N 1s	3.6	3.29	2.25	3.17
Fe 2p	0.39	0.24	0.21	0

Table S5 Assignments of N species for different samples.

N species (at%)	$NCA_{C\text{-}Zn}$/Fe	NCA_C/Fe	CA_C/Fe	CA_C
Pyridinic N	0.51	0.52	0.22	0.47
Fe-N	0.44	0.29	0.04	0
pyrrolic N	0.54	0.12	0.06	0
graphitic N	1.79	1.84	1.54	1.95
oxidized N	0.31	0.52	0.38	0.75

2 Figures of morphological characterizations

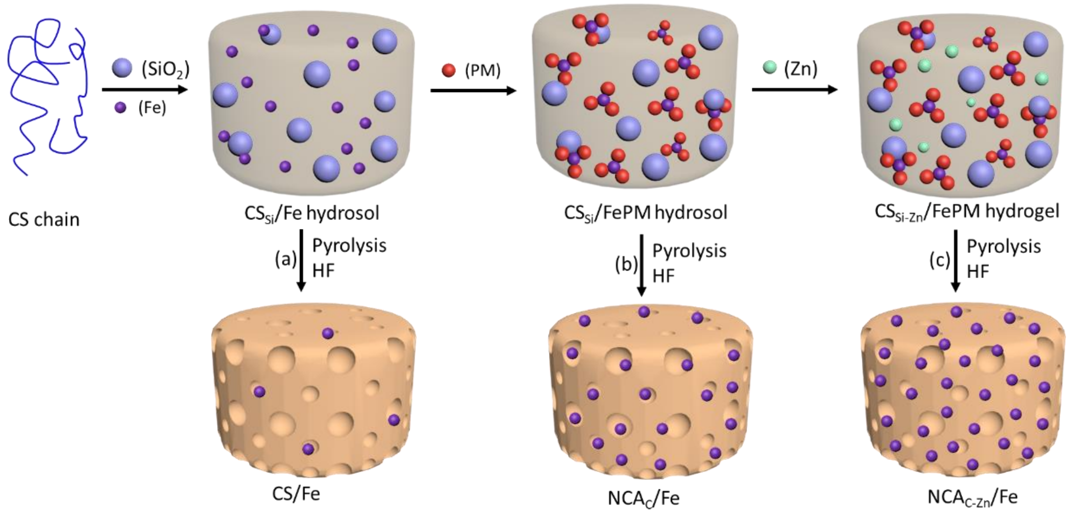

Figure S1 Schematic illustration of the preparation of $NCA_{C\text{-}Zn}$/Fe carbon aerogels. Zn ions can induce the hydrogelation of CS hydrosol to form a uniform 3D network, which facilitates the generation of nanowrinkles.

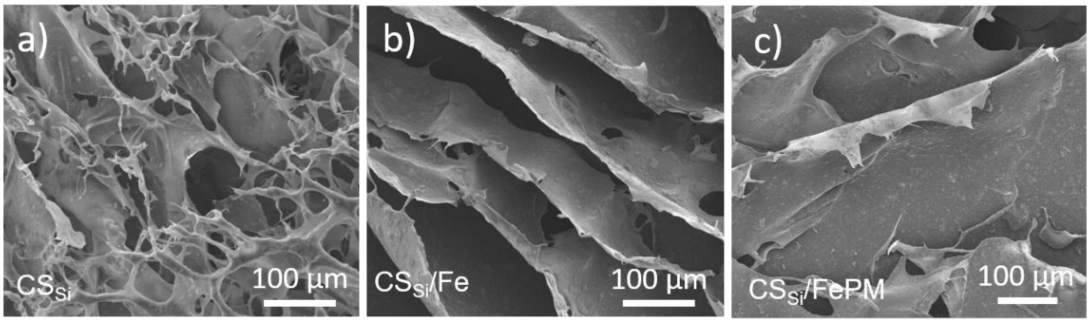

Figure S2 SEM images of CS_{Si}, CS_{Si}/Fe and CS_{Si}/FePM.

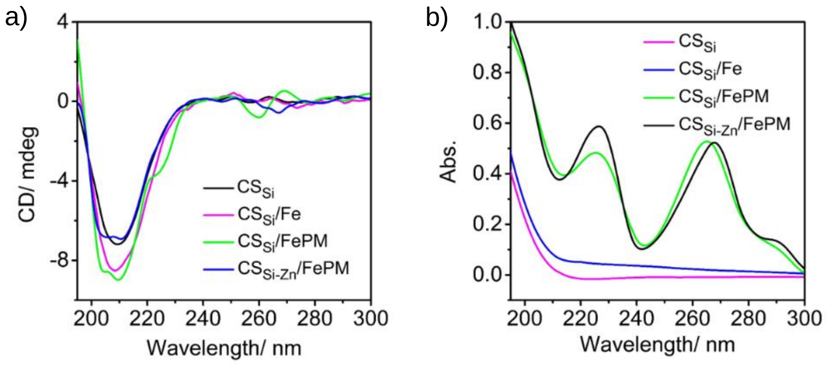

Figure S3 (a) CD and (b) UV-Vis spectra of the series of biomass-derived hydrogels.

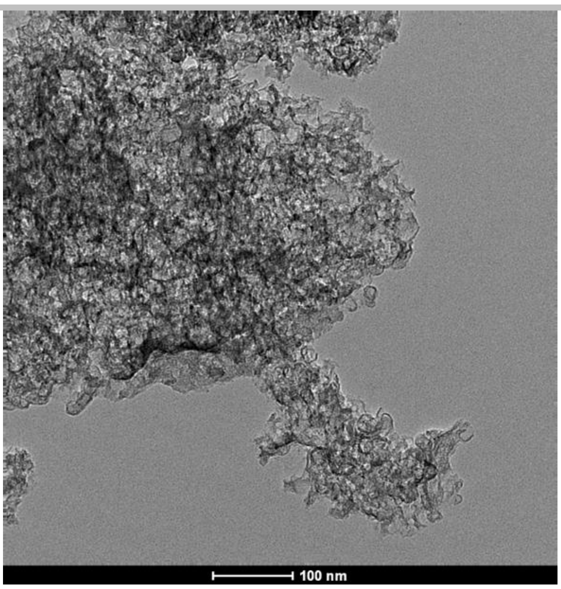

Figure S4 TEM image of $NCA_{C\text{-}Zn}$/Fe aerogel.

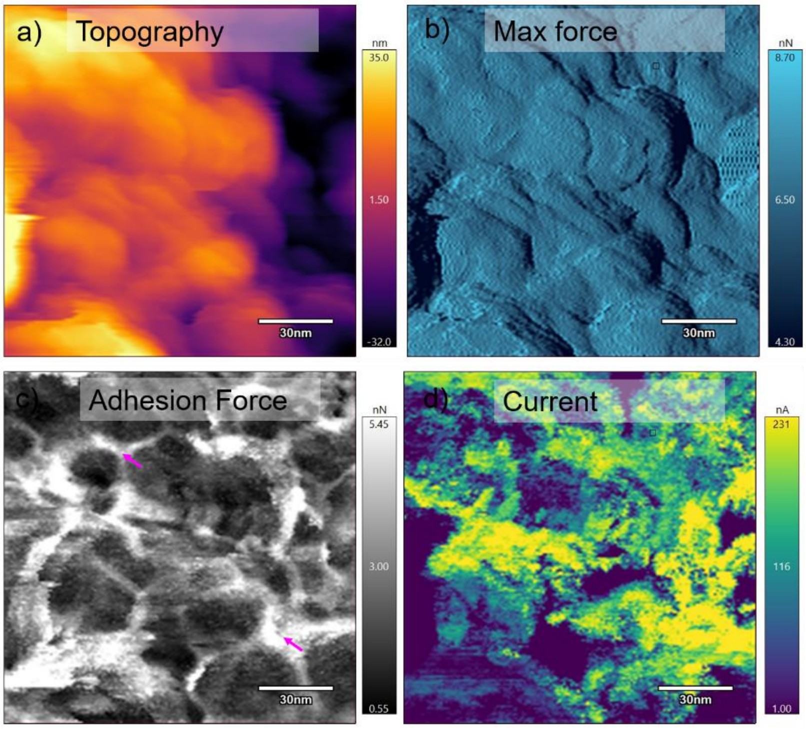

Figure S5 AFM images of $NCA_{C\text{-}Zn}$/Fe aerogels: (a) Topography image. (b) Max force image. (c) adhesion force image, and (d) current flow image.

In order to examine the mechanical and electrical properties of activated porous carbon, we utilized the Fast Force Mapping technique from an Oxford Instruments Asylum Cypher S AFM housed in an Ar gas filled glove box. The O_2 and H_2O levels within the glove box were each below 0.1 ppm. Topography, maximum force, adhesion force, and current flow were measured using the Fast Force mapping technique on our sample at each pixel as the surface was scanned. To perform the current flow measurement component of our experiments we constructed a conductive and flat supporting substrate

for the activated porous carbon. This substrate consisted of an 80 nm thick graphite flake (acquired via mechanical exfoliation of HOPG) resting a SiO_2/Si wafer. A Cr/Au electrode was deposited onto the graphite flake by using thermal evaporation through a shadow mask. A suspension of the activated porous carbon was then drop cast onto the graphite platform, and a bias V_b = 800 mV was applied between the electrode and the conductive AFM tip (Asyelec 75 KHz, coated with Ti/ Ir). In each pixel of the Fast Force Map scan, the AFM tip is pressed into the sample until it applied a force of ~7 nN, as determined by the spring constant ($k = 2.8\ N/m$) and desired deflection of the AFM cantilever. Four quantities were extracted simultaneously from the deflection curve of the AFM cantilever: (1) the z-position at which the cantilever began deflection (topography); (2) the maximum deflection of the cantilever as it is pressed into the sample (max force); (3) the negative deflection incurred by the tip as it is retracted from the sample (adhesion force); and (4) the maximum current flowing through the sample (current).

Figure S5 shows four scans of the drop cast activated porous carbon that were taken using the methods described above. Panel (a) is a 150 nm square scan of the topographic height of porous carbon. The height in this scan has a range of 70 nm and is depicted using a 3-tone color scale with low, medium and high features colored in black, pink and yellow, respectively. Round, ~10 nm wide features clearly protrude upward ~10 nm in this topographic scan. Figure S5 (b) depicts the corresponding max force scan, with a range of 4.3 nN (black) to 8.7 nN (aqua). Dark regions outline the right side of round regions in (a), light areas highlight the left side of the same features. Figure S5 (c) portrays the adhesion force of the same region in (a,b), with a range of 0.55 nN (black) to 5.45 nN (white). White regions form an intricate network of edges through this scan, connecting at nodes with typically three edges forming trigonally symmetric vertices. These features are indicated by pink arrows. Additionally, darker areas appear to be bordered by these high adhesion or “sticky” nodes. Panel (d) shows the maximum current flowing through the porous carbon sample when the tip is brought into contact. A 3-tone color scale with ~0 nA, ~100 nA and ~200 nA depicted in navy blue, teal and yellow, respectively is used to indicate the measured current at each pixel within the scan window. The current shows a large variation over the scan window, with roughly equal areas of high and low current. Interestingly, the regions of high or low current exhibit structure that varies on the order of ~ 10 nm.

3 Figures of structural characterizations

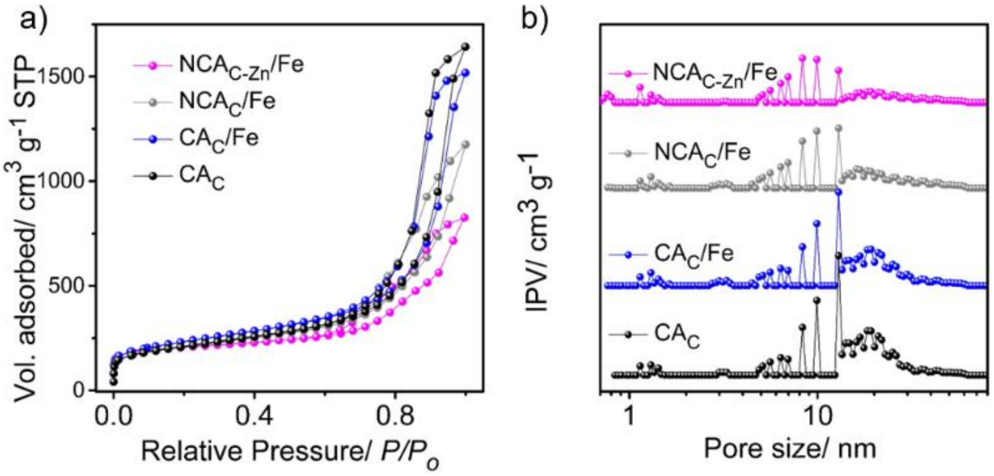

Figure S6 (a) N_2 absorption-desorption isotherm and (b) pore size distribution of CA_C, CA_C/Fe, NCA_C/Fe and NCA_{C-Zn}/Fe.

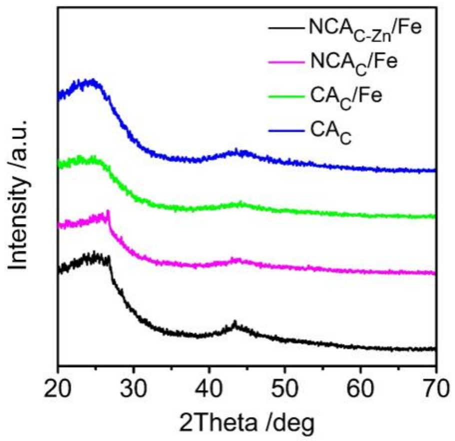

Figure S7 XRD patterns of CA_C, CA_C/Fe, NCA_C/Fe and NCA_{C-Zn}/Fe.

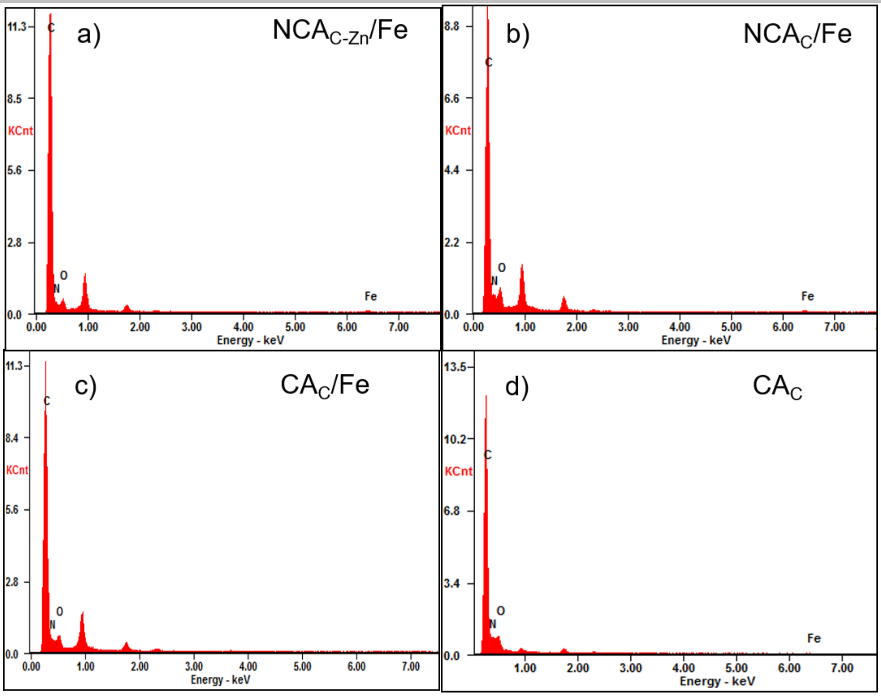

Figure S8 EDS profiles of CA$_C$, CA$_C$/Fe, NCA$_C$/Fe and NCA$_{C-Zn}$/Fe. The results are summarized in Table S2.

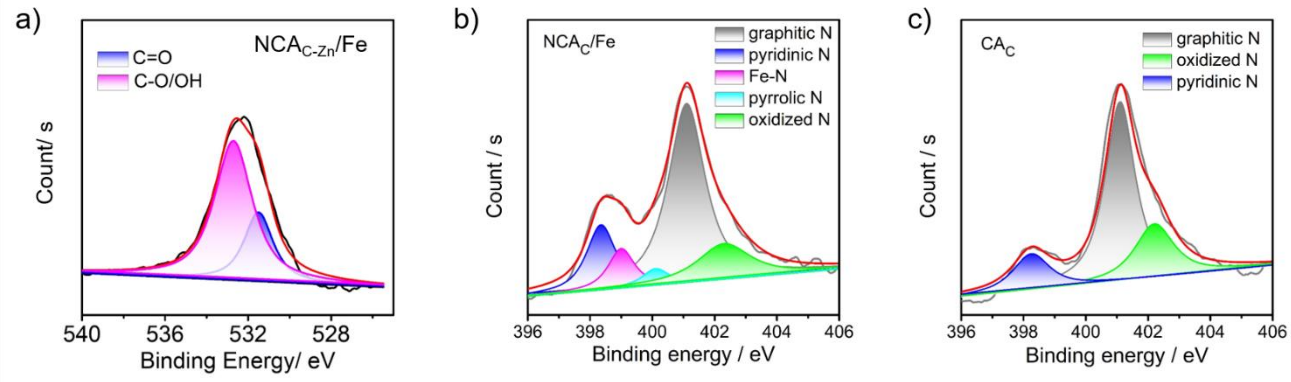

Figure S9 (a) XPS scans of O 1s electrons of NCA$_{C-Zn}$/Fe. XPS scans of the N 1s electrons of (b) NCA$_C$/Fe and (c) CA$_C$. The results are summarized in Table S4.

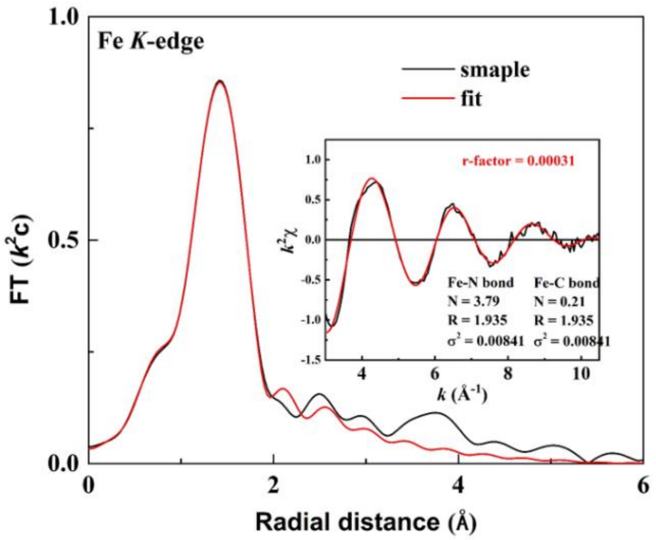

Figure S10 EXAFS fitting curves for NCA_{C-Zn}/Fe. Inset is the corresponding K-space profiles.

4 Figures of DFT

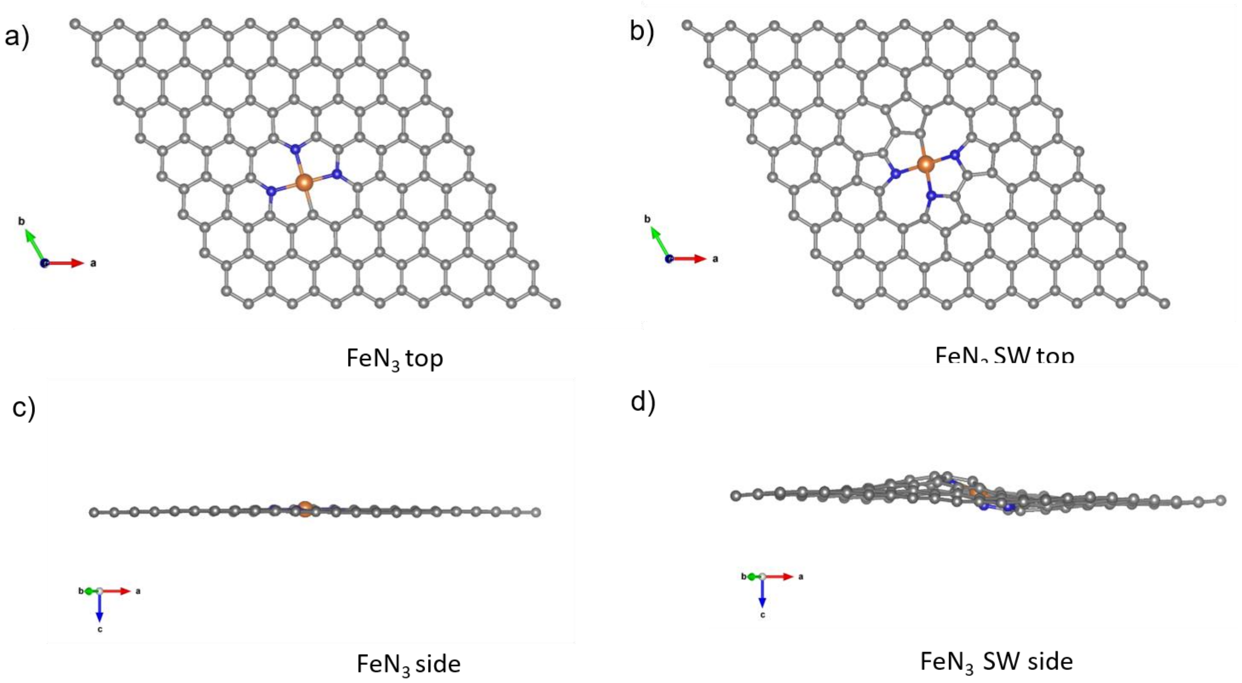

Figure S11 Top view of (a) normal FeN_3 and (b) FeN_3 SW moieties in graphene; Side view of (c) normal FeN_3 and (d) FeN_3 SW moieties.

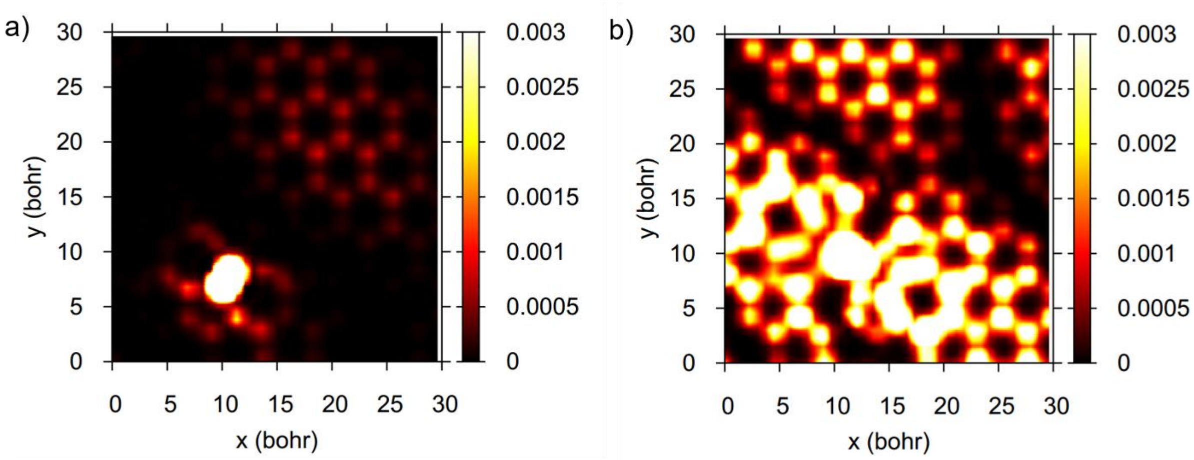

Figure S12 Simulated STM image (at a bias of 1.0 V) of (a) normal FeN_4 and (b) FeN_4 SW doped graphene sheets.

5 Figures of electrocatalytic measurements

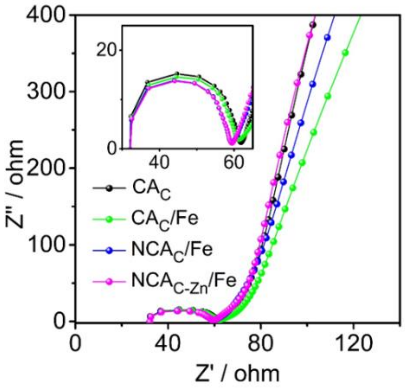

Figure S13 EIS spectra of CA_{C}, CA_{C}/Fe, NCA_{C}/Fe and $NCA_{C\text{-}Zn}$/Fe.

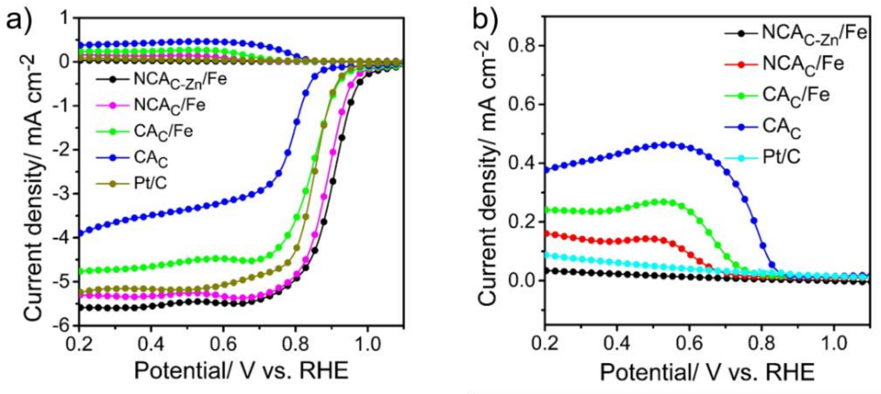

Figure S14 (a) RRDE polarization curves of CA_{C}, CA_{C}/Fe, NCA_{C}/Fe, $NCA_{C\text{-}Zn}$/Fe, as well as Pt/C at 1600 rpm in 0.1 M KOH. Potential scan rate 5 mV s^{-1}. (b) is the zoom-in of the ring currents in (a).

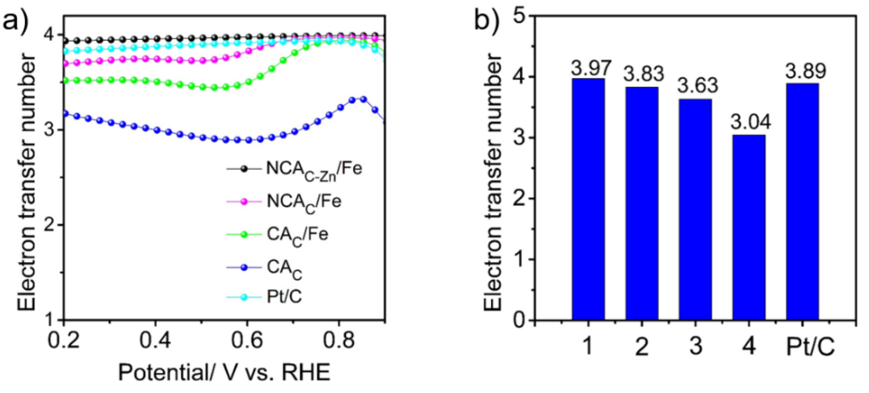

Figure S15 (a) Electron transfer numbers of the biomass-derived carbon aerogels and Pt/C at different potentials. (b) Average electron transfer numbers of CA_{C} (4), CA_{C}/Fe (3), NCA_{C}/Fe (2), $NCA_{C\text{-}Zn}$/Fe (1) and Pt/C.

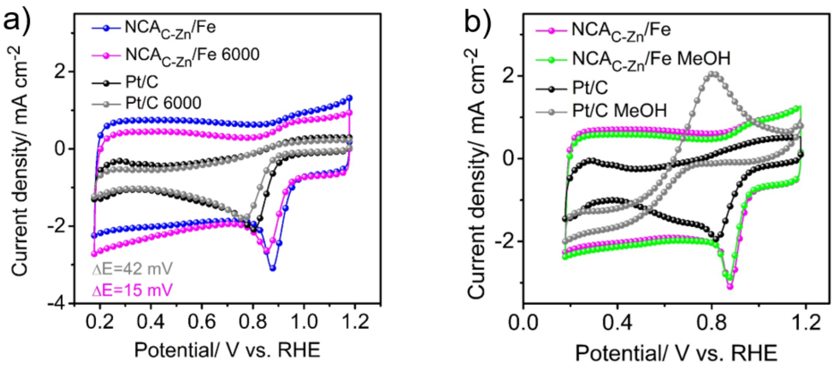

Figure S16 (a) Durability tests of NCA$_{C\text{-}Zn}$/Fe and Pt/C; scan rate: 50 mV s^{-1}, medium: O_2-saturated 0.1 M KOH. (b) CV curves of the NCA$_{C\text{-}Zn}$/Fe and commercial Pt/C as ORR catalysts in the absence and presence of 1 M MeOH.

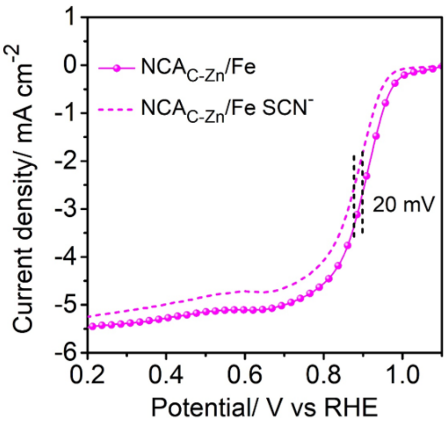

Figure S17 ORR polarization curves of NCA$_{C\text{-}Zn}$/Fe in 0.1 M KOH with or without 10 mM KSCN.

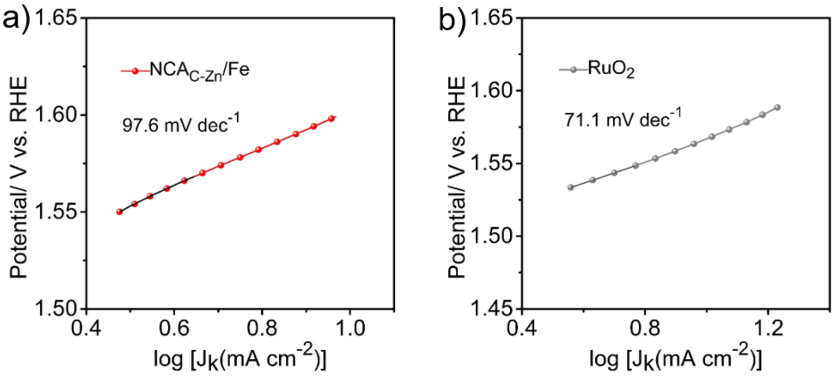

Figure S18 OER Tafel plots of (a) NCA$_{C\text{-}Zn}$/Fe and (b) RuO_2.

6 Figures of metal-air tests measurements

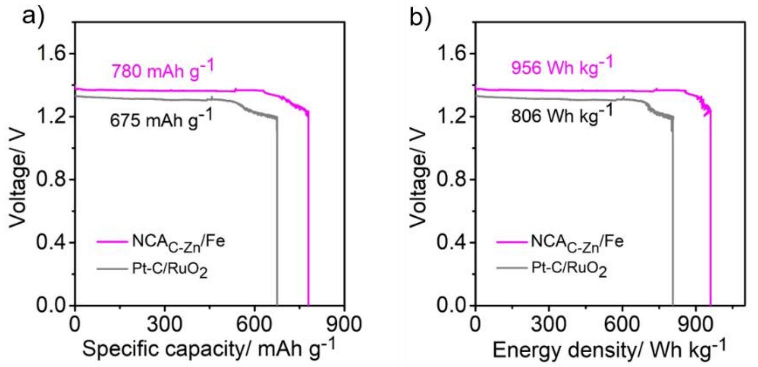

Figure S19 (a) Specific capacity and (b) energy density of a Zn-air battery using $NCA_{C\text{-}Zn}$/Fe or Pt/C-RuO_2 as the air cathode catalysts.

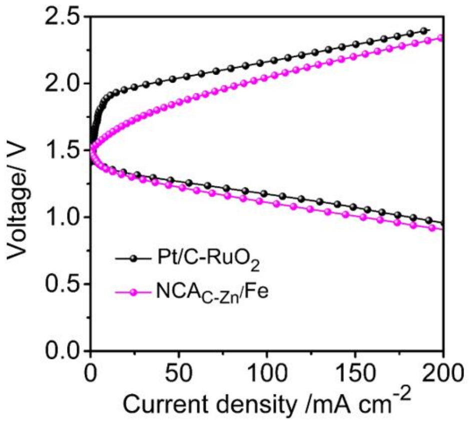

Figure S20 Charge-discharge tests of of a Zn-air battery using $NCA_{C\text{-}Zn}$/Fe or Pt/C-RuO_2 as the air cathode catalysts.
